# Comparison of Diabetes Mellitus Risk Factors in Mexico in 2003 and 2014

**DOI:** 10.3389/fnut.2022.894904

**Published:** 2022-06-30

**Authors:** Guillermo F. López Sánchez, Rubén López-Bueno, Carlos Villaseñor-Mora, Shahina Pardhan

**Affiliations:** ^1^Division of Preventive Medicine and Public Health, Department of Public Health Sciences, School of Medicine, University of Murcia, Murcia, Spain; ^2^Department of Physical Medicine and Nursing, University of Zaragoza, Zaragoza, Spain; ^3^Division of Sciences and Engineering, University of Guanajuato, Guanajuato, Mexico; ^4^Vision and Eye Research Institute, School of Medicine, Faculty of Health, Education, Medicine and Social Care, Anglia Ruskin University, Cambridge, United Kingdom

**Keywords:** diabetes, prevalence, risk factors, Mexico, WHO surveys

## Abstract

**Objective:**

The prevalence of diabetes mellitus in Mexico is very high. This study aimed to compare the risk factors of diabetes mellitus in Mexican adults in 2003 and in 2014.

**Methods:**

This study had a repeated cross-sectional design. Data from the World Health Organization (WHO) Study on global AGEing and adult health (SAGE) from Mexico (Wave 0, 2003, and Wave 2, 2014) were compared. Self-reported diabetes mellitus (outcome) was evaluated with the yes/no question: “Have you ever been diagnosed with diabetes mellitus (high blood sugar)?” Bivariate analyses and multivariable logistic regression analyses adjusted for potential risk factors were conducted.

**Results:**

In 11 years (2003–2014), the prevalence of self-reported diabetes mellitus in Mexican adults increased by 2.6 times in those younger than 50 years (2003: 2.1%; 2014: 5.5%) and by 1.9 times in those ≥50 years (2003: 12.7%; 2014: 24.2%). In 2003, the risk factors associated with diabetes mellitus were female sex (OR 1.344, 95% CI 1.176–1.536), age ≥50 years (OR 6.734, 95% CI 5.843–7.760), being overweight (OR 1.359, 95% CI 1.175–1.571), obesity (OR 1.871, 95% CI 1.583–2.211), and lower physical activity of <600 MET-minutes/week (OR 1.349, 95% CI 1.117–1.630). In 2014, the exposure characteristics significantly associated with diabetes mellitus were female sex (OR 1.244, 95% CI 1.025–1.511), older age ≥50 years (OR 4.608, 95% CI 3.260–6.515), being overweight (OR 1.649, 95% CI 1.305–2.083), obesity (OR 1.778, 95% CI 1.398–2.261), and in those who had not attended/completed primary school (OR 1.360, 95% CI 1.042–1.773).

**Conclusion:**

The prevalence of diabetes mellitus in Mexico significantly increased from 2003 to 2014. Female sex, age older than 50 years, and being overweight or obese were significant risk factors in both 2003 and 2014. Not having completed primary school was a new significant risk factor in 2014. Public health policies and strategies should prioritize decreasing the high levels of overweight and obesity, and improve health literacy in Mexico.

## Introduction

The global prevalence of diabetes mellitus in 2019 was ~463 million in adults aged 20–79 years, which is predicted to increase to over 700 million by 2045 (1). The health expenditure for people living with diabetes mellitus is assumed to be twice that of people without the disease, and was reported to be USD 760.3 billion globally in 2019 (1).

Diabetes mellitus is particularly important in Mexico, as it has the 6th highest number of adults with the disease (12.8 million in 2019) in the world, which is predicted to increase to 22.3 million by 2045 (1). The Mexican government implemented a programme (El Sistema de Vigilancia Epidemiológica Hospitalaria de Diabetes Mellitus Tipo 2, SVEHDMT2) to monitor hospital attendance by people suffering from type 2 diabetes mellitus (2). This predicts that by 2030 Mexico will spend the equivalent of 6% of GDP on hospital attendance due to diabetes mellitus. The programme also suggests that males aged 55–59 years old and females aged 60–64 years old are at higher risk of diabetic complications including neuropathy, arterial peripheral disease and nephropathy, with hospitalization increasing with age (2). Diabetes mellitus-related infections in the lower limb are the most common cause of hospitalization, and these can result in amputation, which in turn is the most common cause of death in these patients in Mexico. Nutritional and ophthalmologic monitoring for diabetic patients has not been implemented in over 64% of people attending hospitals. Additionally, the prevalence of diabetes mellitus has been shown to be higher in Hispanics/Latinos compared to non-Hispanic whites, due to both genetic and lifestyle risk factors (3).

Previous cross-sectional studies using Mexican national surveys indicate that the prevalence of diabetes mellitus is increasing: 7.5% in 2000 (4), 9.2% in 2012 (5), 13.7% in 2016 (6), 14.7% in 2018 (7), and 16.9% in 2021 (8). It is now estimated that one in two children will develop diabetes mellitus in the future due to weight and lack of physical activity unless public health policies change (9). In 2019 diabetes was the second highest cause of death in Mexico (10), in 2020 there were 148,437 diabetes-related deaths (11), and in 2021 there were 184,384 diabetes-related deaths in adults aged 20–79 years old, with a proportion of diabetes-related deaths in people under 60 years of 9.9% (12).

Diabetes mellitus should therefore be a major public health priority in Mexico, and prevention and good management is paramount (13). To be able to adequately prevent and manage diabetes mellitus in Mexico, it is important to ascertain whether the risk factors that present a significant association with the high prevalence have changed over time. This would enable policy makers to determine whether their strategies have worked in reducing the prevalence of diabetes mellitus, and to address the strategies that have not worked.

It is known that several modifiable and non-modifiable factors can have an important impact in the risk of diabetes mellitus development (14), including BMI, age, physical exercise, nutrition, tobacco or alcohol consumption, etc. However, there are discrepancies in the literature, highlighting the need for more research (15). For example, smoking or alcohol consumption have been shown to be protective factors in some studies and risk factors in others. In addition, the strength of the associations between risk/protective factors and diabetes mellitus varies in different studies (16–19).

Previous studies have examined the prevalence of diabetes mellitus and associated risk factors in Mexico (4, 6, 7), showing significant associations with age, education, access to and type of health system, living in a rural area, and BMI; however there are no studies comparing significant risk factors of diabetes mellitus in Mexico over an 11 year period. Temporal information is essential for the development of targeted health policy strategies, especially since an incremental trend in the economic burden of diabetes mellitus in Mexico is predicted (20). This study aims to compare the prevalence of diabetes mellitus between 2003 and 2014, using the data of the WHO's Study on Global Aging and Adult Health, and to examine the risk/protective factors among Mexican adults.

## Methods

### The Surveys

This study had a repeated cross-sectional design. We used data from two surveys of the World Health Organization (WHO): the World Health Survey (WHS), also called SAGE Wave 0, and the WHO Study on global AGEing and adult health (SAGE) Wave 2. Ethical approval was not required, since the data used are public and anonymized.

World Health Survey, or SAGE Wave 0 (https://www.who.int/data/data-collection-tools/study-on-global-ageing-and-adult-health), was implemented by the WHO in 2002–2004 in partnership with 70 countries to generate information on the health of adult populations and health systems. It was launched by the WHO to strengthen national capacity to monitor critical health outcomes and health systems through the fielding of a valid, reliable, and comparable household survey instrument. Details of the WHS methodology have been published previously (21–23). In brief, the WHS was implemented in countries selected to represent all regions of the world and study samples were nationally representative and probabilistically selected. Sampling weights were generated and adjusted for the population distribution with final post-stratification corrections for non-response. This survey includes individuals aged 18+ years.

Study on global AGEing and adult health (https://apps.who.int/healthinfo/systems/surveydata/index.php/catalog/whs/about) Wave 2 was undertaken in China, Ghana, India, Mexico, Russia, and South Africa between 2014 and 2015. Details of the SAGE survey methodology have been published previously (24). In brief, in order to obtain nationally representative samples, a multistage clustered sampling design method was used. The sample consisted of adults aged ≥18 years with oversampling of those aged ≥50 years. To be able to adequately compare the data of both surveys, despite the oversampling, the age cutoff established in the analysis was 50 years, dividing the sample in those <50 years and those ≥50 years. Face-to-face interviews were conducted by trained interviewers using a questionnaire. According to the United Nations Statistical Division, sampling weights were prepared in order to adjust for the structure of the population.

The present study analyzed Mexican data in these two surveys. In WHS, there were 38,746 Mexican adults (age range 18–106 years, average age 41.00 ± 16.74 years, 57.73% female), of which 1,259 indicated that they suffered from diabetes mellitus (age range 18–96 years, average age 57.19 ± 14.06 years, 63.30% female). In SAGE, there were 5,908 Mexican adults (age range 18–98 years, average age 61.72 ± 15.13 years, 60.00% female), of which 986 self-reported diabetes mellitus (age range 19–94 years, average age 65.70 ± 9.78 years, 64.50% female).

### Outcome Variable

Diabetes mellitus was evaluated through a self-reported lifetime diagnosis with the yes/no question: “Have you ever been diagnosed with diabetes mellitus (high blood sugar)?” The validity and high accuracy of self-reported diagnosis of diabetes mellitus has been confirmed by previous research (25–27) and previous studies using WHO data have also used this question to evaluate diabetes mellitus (28–30).

### Exposure Variables

Exposure variables were chosen based on those reported in the literature, including age, sex, education level, smoking, Body Mass Index (BMI), marital status, alcohol consumption, fruit and vegetable consumption, and physical activity level. Age was categorized according to the WHO definition of older adults (31) as <60 years and ≥60 years. BMI was calculated using self-reported weight and height, and was classified as (32): <25.0 kg/m^2^ (underweight/normal weight), 25.0–29.9 kg/m^2^ (overweight) and ≥30.0 kg/m^2^ (obese). The marital status of the participants was classified as never married/separated/divorced/widowed (categorized as living alone) or currently married/cohabiting (not living alone). Highest level of education completed was divided into three categories: less than primary school/primary school completed, secondary school completed/high school (or equivalent) completed, and college/pre-university/university completed/post-graduate degree completed. Smoking was assessed by asking the participants: “Do you currently use (smoke, sniff or chew) any tobacco products such as cigarettes, cigars, pipes, chewing tobacco or snuff?” and categorized as: daily; yes, but not daily; no, not at all. Alcohol consumption was evaluated with the question “Have you ever consumed a drink that contains alcohol (such as beer, wine, spirits, etc.)?” and the response options were yes and never. Consumption of fruits and vegetables was evaluated with the questions “How many servings of fruit do you eat on a typical day?” and “How many servings of vegetables do you eat on a typical day?” and the sample was classified in two groups: <5 servings/day and ≥5 servings/day (33, 34). The level of physical activity was measured with two valid and reliable instruments: WHS used the International Physical Activity Questionnaire (IPAQ) Short Form (35, 36) and SAGE used the Global Physical Activity Questionnaire (GPAQ) (37). The physical activity unit of the two instruments was MET-minutes/week Metabolic Equivalent of Task (MET). The formula of IPAQ to calculate the total amount of physical activity in MET-minutes/week was: sum of Walking + Moderate + Vigorous MET-minutes/week scores (38). The formula of GPAQ for the calculation of the total level of physical activity in MET-minutes/week was: sum of the total MET minutes of physical activity carried out in each setting (work, travel to and from places, and recreational activities) (39). The cutoff value used for physical activity was 600 MET-minutes/week, classifying the participants in two groups according to the international physical activity recommendations (38–40).

### Statistical Analysis

The software used to statistically analyze the data was the Statistical Package for the Social Sciences 23.0 (IBM, NY, USA). Diabetes mellitus prevalence (frequencies and percentages) in Mexican adults was analyzed in the two waves (0 and 2) and according to the exposure variables (Table 1). The exposure variables that presented significant differences with chi-square analyses were used in the multivariate regression models (Table 2), except for tobacco, due to missing data. Multivariable logistic regression served to examine the association between exposure variables (i.e., risk factors for diabetes mellitus) and diabetes mellitus (outcome). The Wave 0 model (WHS) was adjusted for sex, age, BMI, marital status, education and physical activity, while the Wave 2 model (SAGE) was adjusted for sex, age, BMI, education, alcohol and physical activity. All variables were included in the models as categorical variables. Results from the logistic regression analyses are presented as odds ratios (ORs) with 95% confidence intervals (CIs) in Table 2. The missing data in people with diabetes mellitus in 2003 were: BMI (*n* = 35; 2.78%) and physical activity (*n* = 138; 10.96%). Missing values in participants reporting diabetes mellitus in 2014 were: BMI (*n* = 134; 13.59%), education (*n* = 147; 14.91%), smoking (*n* = 750; 76.10%) and physical activity (*n* = 341; 34.6%). As tobacco had such a high rate of missing data, firm conclusions could not be made regarding this variable. Complete-case analysis was carried out. *P* < 0.05 was set as the level of statistical significance.

**Table 1:** Prevalence of diabetes mellitus (outcome) in Mexican adults, by year and exposure variables.

**Variables**	**Categories**	**2003** **(*n* = 38,746)**	**2014** **(*n* = 5,908)**	**Ratio% (2014/2003)**
Overall	–	1,259 (5.2)	986 (21.1)	4.1
Sex^2003, 2014^	Females (*n* 2003 = 22,369; *n* 2014 = 2,799)	797 (5.7)	636 (22.7)	4.0
	Males (*n* 2003 = 16,377; *n* 2014 = 1,866)	462 (4.6)	350 (18.8)	4.1
Age^2003, 2014^	<50 years (*n* 2003 = 27,736; *n* 2014 = 762)	352 (2.1)	42 (5.5)	2.6
	≥50 years (*n* 2003 = 11,009; *n* 2014 = 3,903)	907 (12.7)	944 (24.2)	1.9
BMI^2003, 2014^	<25.0: Underweight/normal weight (*n* 2003 = 11,042; *n* 2014 = 1,040)	422 (3.8)	152 (14.6)	3.8
	25.0–29.9: Overweight (*n* 2003 = 8,700; *n* 2014 = 1,660)	491 (5.6)	375 (22.6)	4.0
	≥30.0: Obesity (*n* 2003 = 3,769; *n* 2014 = 1,375)	311 (8.3)	325 (23.6)	2.8
Marital status^2003^	Never married, separated/divorced, widowed (*n* 2003 = 12,223; *n* 2014 = 1,801)	448 (5.8)	380 (21.1)	3.6
	Currently married, Co-habiting (*n* 2003 = 26,523; *n* 2014 = 2,864)	811 (4.9)	606 (21.2)	4.3
Education^2003, 2014^	≤ Primary school (*n* 2003 = 5,437; *n* 2014 = 2,568)	321 (9.1)	602 (23.4)	2.6
	Secondary school completed, high school (or equivalent) completed (*n* 2003 = 33,140; *n* 2014 = 894)	931 (4.5)	136 (15.2)	3.4
	College, pre-university, university, or post-graduate degree completed (*n* 2003 = 169; *n* 2014 = 568)	7 (7.3)	101 (17.8)	2.4
Tobacco^2014^	Daily (*n* 2003 = 3,179; *n* 2014 = 303)	90 (5.1)	53 (17.5)	3.4
	Yes, but not daily (*n* 2003 = 5,917; *n* 2014 = 181)	161 (4.8)	28 (15.5)	3.2
	No, not at all (*n* 2003 = 29,650; *n* 2014 = 642)	1,008 (5.3)	155 (24.1)	4.5
Alcohol^2014^	Yes (*n* 2003 = 18,364; *n* 2014 = 2,574)	598 (5.3)	515 (20.0)	3.8
	Never (*n* 2003 = 20,382; *n* 2014 = 2,091)	661 (5.1)	471 (22.5)	4.4
Fruit & vegetables	<5 servings/day (*n* 2014 = 3,948)	–	845 (21.4)	–
	≥5 servings/day (*n* 2014 = 715)	–	141 (19.7)	–
Physical activity^2003, 2014^	<600 MET-minutes/week (*n* 2003 = 3,363; *n* 2014 = 732)	147 (7.9)	146 (19.9)	2.5
	≥600 MET-minutes/week (*n* 2003 = 32,276; *n* 2014 = 2,629)	974 (4.7)	499 (19.0)	4.0

*Variables that were significant in bivariate analysis in 2003 and 2014 are indicated*.

*Values expressed in Frequencies (Valid%). “Valid percent” is the percent when missing data are excluded from the calculations*.

*^2003^Significant differences in the prevalence of diabetes mellitus in 2003*.

*^2014^Significant differences in the prevalence of diabetes mellitus in 2014*.

*Chi-square tests (χ2) were used to calculate significant differences between groups*.

*Significant exposure variables predicting diabetes mellitus in χ2 tests were included in the regression analyses (Table 2)*.

**Table 2:** Associations between exposure variables and diabetes mellitus (outcome) in Mexican adults, estimated by multivariable logistic regression (by year).

**Variables**	**Categories**	**2003**	**2014**
Sex ^2003, 2014^	Females	1.344 (1.176–1.536)***	1.244 (1.025–1.511)*
	REF: Males	1.0	1.0
Age ^2003, 2014^	REF: <50 years	1.0	1.0
	≥50 years	6.734 (5.843–7.760)***	4.608 (3.260–6.515)***
BMI ^2003, 2014^	REF: <25.0: Underweight/normal weight	1.0	1.0
	25.0–29.9: Overweight	1.359 (1.175–1.571)***	1.649 (1.305–2.083)***
	≥30.0: Obesity	1.871 (1.583–2.211)***	1.778 (1.398–2.261)***
Marital status ^2003^	Never married, separated/divorced, widowed	1.135 (0.990–1.301)	–
	REF: Currently married, Co-habiting	1.0	–
Education^2003, 2014^	≤ Primary school	0.821 (0.320–2.102)	1.360 (1.042–1.773)*
	Secondary school, High school (or equivalent) completed	0.815 (0.320–2.073)	1.167 (0.848–1.607)
	REF: College, pre-university, university, or post-graduate degree completed	1.0	1.0
Alcohol^2014^	Yes	–	0.955 (0.791–1.153)
	REF: Never	–	1.0
Fruit and vegetables	<5 servings/day	–	–
	REF: ≥5 servings/day	–	–
Physical activity^2003, 2014^	<600 MET-minutes/week	1.349 (1.117–1.630)**	1.037 (0.818–1.315)
	REF: ≥600 MET-minutes/week	1.0	1.0

Values expressed in Odds Ratio (95% Confidence Interval). ^*^P < 0.05, ^**^P < 0.01, ^***^P < 0.001. REF, reference category.

*^2003^Logistic regression analyses were adjusted for sex, age, BMI, marital status, education and physical activity*.

*^2014^Logistic regression analyses were adjusted for sex, age, BMI, education, alcohol and physical activity*.

## Results

Overall diabetes mellitus prevalence in Mexican people <50 years was 2.1% in 2003 and 5.5% in 2014, showing a 2.6 times increase. In those aged ≥50 years, the overall prevalence was 12.7% in 2003 and 24.2% in 2014, showing a 1.9 times increase. In 2003, unadjusted analyses (Table 1) show the exposure factors that were significantly (*P* < 0.05) associated with a higher prevalence of diabetes mellitus as: female sex (5.7%), age ≥ 50 years (12.7%), obesity (8.3%), living alone (never married/separated/divorced/widowed) (5.8%), ≤ primary education (9.1%) and <600 MET-minutes/week of physical activity (7.9%). In 2014, the exposure characteristics significantly associated with a higher prevalence of diabetes mellitus were: female sex (22.7%), age ≥ 50 years (24.2%), obesity (23.6%), ≤ primary education (23.4%), no tobacco consumption (24.1%), no alcohol consumption (22.5%) and <600 MET-minutes/week of physical activity (19.9%).

In 2003, multivariable logistic regression analyses (Table 2) observed female sex (OR 1.344, 95% CI 1.176–1.536), age ≥ 50 years (OR 6.734, 95% CI 5.843–7.760), being overweight (OR 1.359, 95% CI 1.175–1.571), obesity (OR 1.871, 95% CI 1.583–2.211), and <600 MET-minutes/week of physical activity (OR 1.349, 95% CI 1.117–1.630) as being significantly associated with diabetes mellitus. In 2014, the risk factors significantly associated with a higher risk of diabetes mellitus were female sex (OR 1.244, 95% CI 1.025–1.511), age ≥ 50 years (OR 4.608, 95% CI 3.260–6.515), being overweight (OR 1.649, 95% CI 1.305–2.083), obesity (OR 1.778, 95% CI 1.398–2.261), and not having attended or completed primary school (OR 1.360, 95% CI 1.042–1.773).

## Discussion

No previous study has compared the prevalence of diabetes mellitus and its associated risk factors in Mexico over a 11-year period (2003 and 2014). Unlike previous research studying diabetes mellitus prevalence, this study highlights not only the difference in prevalence, but also the significant associated risk factors using data collected with similar WHO methodology.

First of all, it is important to consider that the current overall prevalence of diabetes mellitus in Mexico (16.9%) is higher than the average prevalence in Latin America and the Caribbean (9.9%), and higher than other countries in this area including Brazil (8.8%), Colombia (8.3%), Costa Rica (8.8%), Ecuador (4.4%), Honduras (5.1%), and Venezuela (9.6%) (8). In this context, the current study found that, in Mexican people aged under 50 years, there was a significant increase of 2.6 times in diabetes mellitus prevalence (2003: 2.1%: 2014: 5.5%), while in those older than 50 years the increase was 1.9 times (2003: 12.7; 2014: 24.2%). The prevalence rates shown by the WHO data are comparable with those reported in the literature. In 2000, results from the National Health Survey of Mexico (*n* = 45,294 adults) reported a prevalence rate of 7.5% for diagnosed diabetes mellitus (4), which in 2016 had increased to 13.7% (3,700 adults), showing a 1.83 times increase (6). The differences in absolute prevalence rates are most likely due to differences in data collection, sampling, and thresholds used for clinical measurements at a given period of time.

Whilst it is known that diabetes mellitus is influenced by a number of known risk factors (modifiable and non-modifiable), the influence and impact of these risk factors is likely to vary between countries. Previous research found that diabetes mellitus in Mexico is associated with higher age, lower education, public health system, living in an urban area, and higher BMI (4, 6, 7). Whilst we show some similarities with already known risk factors (older age and BMI), our study shows other risk factors that were significantly associated with diabetes mellitus, including sex difference (females), low education level and low physical activity.

Older age (≥50 years) was significantly associated with a higher risk of diabetes mellitus in 2003 and 2014. This association has been reported in previous studies (41–43), and can be attributed to the aging process affecting pancreas and insulin production, or to other risk factors of age, such as free fatty acids and lipid metabolisms disorders, mitochondrial dysfunction, inflammation, insulin resistance, β-cell dysfunction, metabolic syndrome, or other factors (44). The odds ratio associated with older age decreased from 2003 (OR 6.734) to 2014 (OR 4.608), suggesting an improvement in the risk factor due to older age alone, and the contribution of other risk factors.

The relationship between overweight/obesity and diabetes mellitus is universally known (45, 46), and in this study they were found to be significantly associated in both 2003 and 2014. Tumor necrosis factor-α, plasma leptin, and non-esterified fatty acid levels are all high in obesity and can impact insulin resistance and consequently diabetes mellitus (45). Reports suggest that dietary patterns in Mexico have changed over the past 30 years due to a higher concentration of people living in urban areas, which has led to an increase in consumption of processed foods, simple carbohydrates, and soft drinks (13). The strength of the association between being overweight or obese and diabetes mellitus was similar in 2003 (ORs 1.359 and 1.871) and 2014 (ORs 1.649 and 1.778), showing that the risk of higher BMI is continuing and needs to be addressed.

Female sex was found to be significantly associated with a higher risk of diabetes mellitus in 2003 (OR 1.344, 95% CI 1.176–1.536) and in 2014 (OR 1.244, 1.025–1.511). While these results do not agree with the global sex distribution of diabetes mellitus (9.0% female vs. 9.6% male) reported in 2019 (1), it is consistent with other studies from Mexico within the period 2000–2016: 7.8% female vs. 7.2% male (4); 10.3% female vs. 8.4% male (47); 22.9% female vs. 18.6% male (48). It is possible that this is due to lower access to healthcare amongst women in Mexico, or sex-specific cultural traditions leading to lower prevention rates and inadequate control in women compared to men (49, 50). This needs to be investigated further.

We also found that, in 2014, lower levels of education (primary school or lower) was significantly associated with a higher risk of diabetes mellitus (OR 1.360, 95% CI 1.042–1.773). This has also been reported in the literature (51), suggesting a need to target certain groups to improve health literacy.

Regarding physical activity, less than 600 MET-minutes/week of physical activity was significantly associated with a higher risk of diabetes mellitus in 2003 (OR 1.349, 95% CI 1.117–1.630). The beneficial role of physical activity in the prevention of diabetes mellitus is well-known (52–56). While physical inactivity was not found to be significant in 2014, overweight and obesity were retained as significant in 2014, suggesting strongly that health policy makers in Mexico need to pay attention to these modifiable risk factors.

The diabetes mellitus epidemic in Mexico has been declared a national emergency, and the country is fighting the epidemic *via* the National Strategy for the Prevention and Control of Overweight, Obesity and Diabetes on three fronts: public health, medical care, and fiscal and regulatory policies (57). Diabetes is currently covered by health insurance and there are national diabetes action programmes (58). Government actions to control diabetes include: the development of massive communication programmes to raise awareness of the disease and of the benefits of healthy weight, adequate diet and physical activity; regulation of food distributed in primary schools; launch of massive self-care diabetes campaigns; unification of guidelines and criteria to diagnose and control diabetes; development of self-support groups for diabetic patients; strengthening of knowledge and competences for health personnel and improvement of access to information by the health sector and general population; development of a National Health Card for adults (similar to the child vaccination card), in which criteria and objectives for health risks are prioritized and evaluations of healthy weight, blood sugar, blood pressure and lipids are emphasized (58). Although the government is attempting to reduce the prevalence of diabetes mellitus, the rate is still increasing as evidenced in the literature. Currently, screening is based on an opportunistic strategy with sporadic population base campaigns; Mexican guidelines for the treatment of diabetes exist, but few doctors are familiar with them or apply them; primary care clinics (responsible for the treatment of the majority of the cases) do not have the infrastructure to treat chronic diseases; public awareness of the disease is low and patients often do not understand the treatment goals and do not make necessary lifestyle changes (58).

Strengths of the present research include the national representativeness of the WHO samples of Mexican adults using standardized questionnaires, the adequate distribution by sex and to the wide age range studied. However, this study had also some limitations. The design was cross-sectional which does not allow causality to be established. In addition, diabetes mellitus was measured with self-reported questions. The type of diabetes mellitus (i.e.,1 or 2) was not collected in the survey. In the WHO study (SAGE) only individuals with a valid home address were included, which may have precluded people living in sheltered accommodation, etc., and who could have a higher risk of diabetes mellitus. Finally, the questionnaire IPAQ used to measure physical activity in SAGE Wave 0 was primarily designed to be used in adults <65 years, but in this survey was used on those older than 65 years. However, new studies have indicated that this instrument is adequate and useful to evaluate physical activity among older adults (59).

## Conclusions

According to WHO data, diabetes mellitus prevalence in Mexico doubled from 2003 to 2014 (increase of 2.6 times in those <50 years and 1.9 times in those ≥50 years). Female sex, age older than 50 years, and being overweight or obese were significant risk factors in both 2003 and 2014. Physical inactivity was a significant risk factor only in 2003 and a low education level was a significant risk factor in 2014. While Mexico may have made important strides in overcoming some of the significant risk factors since 2003, attention needs to be given by policy makers to try to reduce the modifiable risk factors, overweight and obesity, and also to improve heath literacy in order to reduce the huge impact of diabetes mellitus in Mexico. We recommend that public health strategies focus specifically on taking action against the growing trend of obesity in Mexico, as this is likely to decrease the incidence of diabetes mellitus in the country.

## Data Availability Statement

Publicly available datasets were analyzed in this study. This data can be found here: https://www.who.int/healthinfo/survey/en/; http://www.who.int/healthinfo/sage/en/.

## Ethics Statement

Ethical review and approval was not required for the study on human participants in accordance with the local legislation and institutional requirements. Written informed consent for participation was not required for this study in accordance with the national legislation and the institutional requirements.

## Author Contributions

All authors listed have made a substantial, direct, and intellectual contribution to the work and approved it for publication.

## Funding

GFLS and RL-B are funded by the European Union—Next Generation EU. SAGE was supported by the U.S. National Institute on Aging through Interagency Agreements OGHA 04034785, YA1323–08-CN-0020, Y1-AG-1005–01, and through research grants R01-AG034479 and R21-AG034263.

## Conflict of Interest

The authors declare that the research was conducted in the absence of any commercial or financial relationships that could be construed as a potential conflict of interest.

## Publisher's Note

All claims expressed in this article are solely those of the authors and do not necessarily represent those of their affiliated organizations, or those of the publisher, the editors and the reviewers. Any product that may be evaluated in this article, or claim that may be made by its manufacturer, is not guaranteed or endorsed by the publisher.
